# Mitochondrial PCR-RFLP Assay to Distinguish *Triatoma brasiliensis macromelasoma* from *Triatoma brasiliensis brasiliensis* Subspecies (Hemiptera: Reduviidae)

**DOI:** 10.1155/2013/305198

**Published:** 2013-12-17

**Authors:** Daniel Pagotto Vendrami, Walter Ceretti-Junior, Marcos Takashi Obara, Mauro Toledo Marrelli

**Affiliations:** ^1^Department of Epidemiology, Faculty of Public Health, Universidade de São Paulo, São Paulo, Avendia Dr. Arnaldo 715, 01246-904, SP, Brazil; ^2^Institute of Tropical Medicine, Universidade de São Paulo, São Paulo, Brazil; ^3^Laboratory of Medical Entomology, Health Surveillance Secretariat, Government Agency for Health, Brasília, DF, Brazil

## Abstract

*Triatoma brasiliensis sensu lato* (s.l.), the main vector of Chagas disease in northeastern Brazil, is a species complex comprising four species, one with two subspecies (*T. brasiliensis brasiliensis*, *T. brasiliensis macromelasoma*, *T. juazeirensis*, *T. sherlocki*, and *T. melanica*), and each taxon displaying distinct ecological requirements. In order to evaluate the genetic relationships among nine *T. brasiliensis* s.l. populations from northeastern Brazil, we analyzed their mitochondrial cytochrome c oxidase subunit 1 sequences and suggested a PCR-RFLP assay to distinguish between *T. b. macromelasoma* and *T. b. brasiliensis* subspecies. All the specimens were morphologically identified as *T. b. brasiliensis*. The resulting phylogenies identified two major clades that are congruent with the geographical populations studied. Based on collection sites and in accordance with type-location, one clade was identified as the subspecies *T. b. macromelasoma.* The second clade grouped *T. b. brasiliensis* populations. Restriction endonuclease sites were observed in the sequences and used in PCR-RFLP assays, producing distinct fingerprints for *T. b. macromelasoma* and *T. b. brasiliensis* populations. The results suggest that these are different species and that gene flow occurs only among *T. b. brasiliensis* populations, possibly associated with human activity in the area.

## 1. Introduction

About 28 million people live in areas at risk of Chagas disease, 11–14.5 million of whom are affected worldwide. *Trypanosoma cruzi,* the pathogen that causes Chagas disease, is found in most South American countries, representing an important cause of heart damage among the economically active population [1]. After a successful chemical control of *Triatoma infestans* (Klugi, 1834), the other main vectors of Chagas causing agent, *Panstrongylus megistus* Burmeister, 1835, *Rhodnius prolixus* Stal, 1859, and *Triatoma brasiliensis sensu lato* Neiva 1911. *T. brasiliensis* kept attracting considerable attention from local entomological surveillance.

*Triatoma brasiliensis sensu lato *(s.l.), found in anthropogenic habitats and considered the main vector in northeast Brazil [2, 3], was recently found to be a species complex that includes *T. b. brasiliensis*, *T. b. macromelasoma *Galvão, 1956*, T. juazeirensis *Costa & Felix, 2007, *T. sherlocki *Papa, Jurberg, Carcavallo, Cerqueira & Barata, 2002, and *T. melanica *Costa et al., 2006. These taxa exhibit wide phenotypic and morphological variability, displaying specific ecological requirements and chromatic patterns [4]. In this respect, accurate species identification is necessary for effective vector control.

The systematic of Triatominae species is based on morphological characters of the adult exoskeleton and male phallic structures [5]. However, insects captured during vector monitoring and control or received for identification and notification are often immature. Although their characteristics are similar to those of adult individuals, they are difficult to distinguish. The morphology of Triatominae species is not well described; with studies on the immature forms performed for only 40 species, eggs and nymphs described for only 20 species, a key to identify nymphs to the species level has yet to be developed. Available keys are useful and partially applicable to other stages, but specific identification of all live forms remains unresolved [6].

Members of the *T. brasiliensis* complex have been distinguished by analyzing isoenzymes [7], mitochondrial DNA sequences [8], and random amplification of polymorphic DNA-RAPD [9]. In the present study we analyzed the barcoding *CO1 *sequences of nine *T. brasiliensis *s.l. populations from northeastern Brazil (States of Pernambuco, Paraíba, and Rio Grande do Norte) in order to identify their genetic relationships. We also conducted a PCR-RFLP assay to distinguish between *T. b. macromelasoma* and *T. b. brasiliensis* subspecies.

## 2. Materials and Methods

### 2.1. Sample Collection

**Figure 1:**
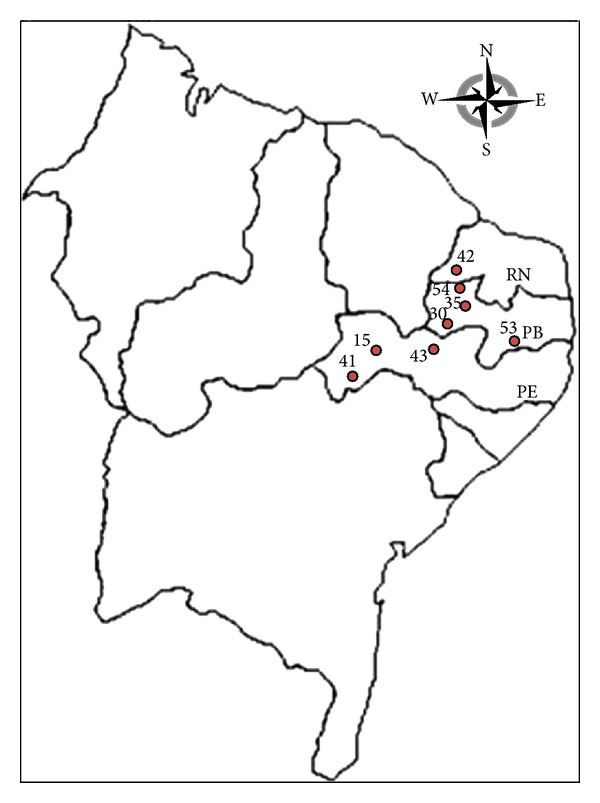
Map of northeast region of Brazil indicating the localities of the *Triatoma brasiliensis* populations studied. Paraíba State: Monteiro (30), Mãe d'água (35), Santa Cruz (54), and São José (53), São Francisco (55). Pernambuco State: Lagoa Grande (41), Salgueiro (15), and Serra Talhada (43). Rio Grande do Norte State: Caicó (42).

Triatominae were collected by surveillance technicians during active inspections in anthropogenic environments (domestic and peridomestic habitats). Live specimens were collected in nine localities in Northeast Brazil (Table 1, Figure 1) using tweezers, flashlights, and PIRISA. Housed in plastic boxes (7 cm diameter × 8 cm high) lined with folded filter paper, the bugs were transported in coolers to the Culicid and Triatomine Laboratory of the Department of Epidemiology, Faculty of Public Health/USP.

Adults were identified as *T. b. brasiliensis* according to the key by Lent and Wygodzinsky [5]. Nymphs were assumed to be *T. brasiliensis* s.l. because, in addition to the difficulty in distinguishing immature Triatominae based on chromatic and morphological characters, comparative material or a key for nymph identification to species level are not available. After morphological identification, DNA was extracted from individuals from the nine localities and sequenced the 520 bp barcode portion of the *CO1* gene (Table 1). Specimens collected in Pernambuco State (even nymphs) were assumed to be *T. b. macromelasoma*, according to Costa et al. [10] and Costa et al. [11].

### 2.2. DNA Extraction and *CO1* Amplification

Genomic DNA was extracted from the legs of 10 individual samples of each population using the Qiagen DNeasy Blood and Tissue Kit (Qiagen, Crawley, United Kingdom) following the manufacturer's protocol. The *CO1* barcode region was amplified from whole genomic DNA using primers LCOI 1490 (5′-GGT CAA CAA ATC ATA AAG ATA TTG G-3′) and HCOI 2198 (5′-TAA ACT TCA GGG TGA CCA AAA AAT-3′) [12].

PCR amplification was carried out in a final volume of 50 *μ*L containing PCR buffer, 0.2 mM of each dNTP, 2.5 mM MgCl_2_, and 1.25 units of *Taq *polymerase. Initial PCR denaturation was at 94°C for 5 min, followed by 40 cycles of denaturation (1 min) at 94°C, annealing (2 min) at 50°C, and extension (2 min) at 72°C. A final extension step at 72°C was performed for 10 min. The amplified DNA was loaded onto 1.5% agarose gel and stained with ethidium bromide. Amplicons were sequenced in both forward and reverse directions using ABI PRISM BigDye Terminator Cycle Sequencing Ready Reaction Kits (Perkin Elmer, Foster City, CA) on an ABI PRISM 3100 Genetic Analyzer/HITACHI.

### 2.3. Analysis of DNA Sequence

Sequences were aligned using Clustal W [13]. One or two representative haplotypes for each population was chosen because of the low variation within populations. Phylogenetic reconstructions were performed by Neighbor Joining and Maximum Likelihood methods (both using the Kimura-3-parameter distance model K81) in MEGA 5.0 [14], and a divergence matrix was constructed under Kimura two parameters (K2P) (Table 2). Maximum Parsimony was carried out using the *branch-and-bound* search option. Phylogenetic analyses included 1000 bootstrap replicates and a *Triatoma sordida CO1* sequence (Genbank acc. no. AF021213) as outgroup. *Triatoma brasiliensis* s.l. *CO1 *sequences were also analyzed using NEBcutter version 2.0 [15] to select appropriate endonuclease enzymes.

### 2.4. Enzyme Restriction Analysis

Individual *CO1 *sequences were PCR-amplified using the above parameters and digested in a 10 *μ*L reaction with *Sty*I (Promega) and *Hinc*II (New England Biolabs, Ipswich, MA) enzymes. The reaction contained 1 *μ*L of 10x buffer, 4 *μ*L deionized water, 4 *μ*L of amplification product, and 1 unit of restriction enzyme. The digestion mixture was incubated at 37°C for 2 h and then resolved on 2.0% agarose gel.

## 3. Results

**Figure 2:**
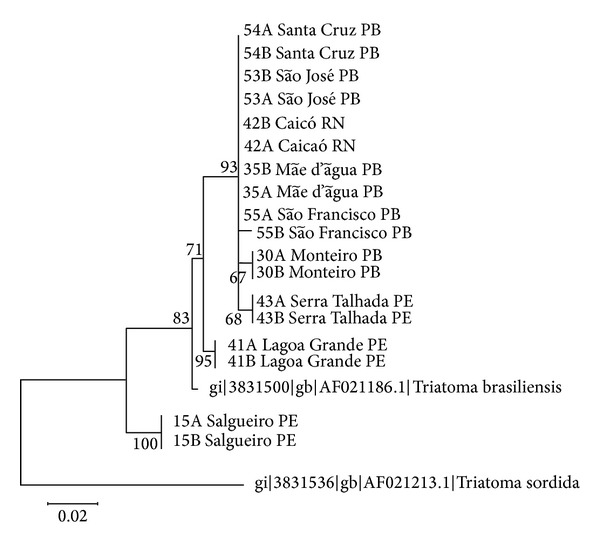
Maximum Likelihood phylogeny generated from *CO1* sequences using the Kimura-2-parameter model with 1000 bootstrap replicates. Sequences were rooted with *T. sordida* (GenBank acc. no.: AF021213).

Phylogenetic trees derived from the Neighbor Joining, Maximum Likelihood, and Parsimony methods showed similar topologies (data shown for ML tree in Figure 2). Since most of *CO1 *sequences were identical into and among the populations, the ML tree was constructed using only two samples of each one. This ML tree and the Nucleotide Distance Matrix (Table 2) indicated sequence divergence of up to 4% between the two main clades. The basal clade, with about 4% divergence from the other populations, consisted of the *Salgueiro* population (15A/B). This population was considered to be *T. b. macromelasoma *due to its high sequence divergence compared to the other populations and because it was collected in its type locality [16]. The second clade, with pairwise distances up to 1%, showed that *Pernambuco* populations are more basal, although those from *Serra Talhada* clustered with *Paraíba* populations likely because of the city's proximity to the Pernambuco-Paraíba border. *Paraíba* populations formed a large cluster that also included the *Rio Grande do Norte* population.

**Figure 3:**
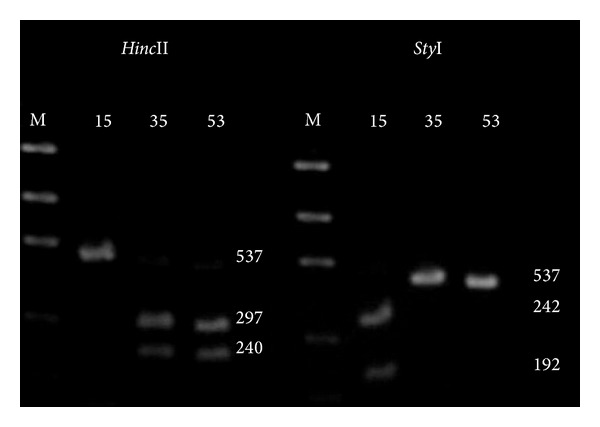
PCR-RFLP fingerprint of *T. brasiliensis* from three localities produced with *Sty*I and *Hinc*II enzymes on 2.0% agarose gel. M: DNA ladder 100 bp (New England Biolabs), 15: *Salgueiro*, 35: *Mãe d'água*, and 53: *São José*.

Based on sequence analysis, a PCR-RFLP assay was performed to differentiate between the subspecies *T. b. macromelasoma* and *T. b. brasiliensis*. PCR fragment digestion using the *Sty*I enzyme produced two restriction fragments (342 bp and 192 bp) in *CO1 *sequences from *Salgueiro* samples and only one fragment in samples of the other eight populations. Conversely, the *Hinc*II enzyme yielded two fragments (297 bp and 240 bp) in all population samples (*n* = 10 samples of each population) except that from *Salgueiro* (Figure 3). *Sty*I and *Hinc*II enzymes therefore produced distinct fingerprints for *T. b. macromelasoma* and *T. b. brasiliensis*, suggesting that they are different subspecies. The molecular protocols described above are a useful tool in the study of populations and cryptic species, contributing to the identification of insect vectors.

## 4. Discussion

The identification of adult Triatominae based on morphological and chromatic pattern is considered relatively easy for most species; however, this is commonly misguided owing to the wide phenotypic variability within this subfamily. For instance, *Triatoma maculata* Erichson, 1848 and *Triatoma pseudomaculata* Corrêa & Espínola, 1964 which were first treated as members of a same species complex due to morphological similarities [5] thereafter proved to be genetically distant [17–19]. *Panstrongylus herreri* Wygodzinsky, 1948 and *Panstrongylus lignarius* Walker, 1873 in turn, were considered to be separate species until Marcilla et al. [20] and Crossa et al. [21] demonstrated that they are the same species, cytogenetically identical with regard to the second internal transcribed spacer. Another difficulty in identifying genera and species of Triatominae is their extensive chromatic variability. The color of some species such as *Rhodnius *sp. (light brown tones) and* Rhodnius nasutus* Stål, 1859 (pinkish tones) seems to be associated with the color of the palm trees they colonize [22], but others such as *Triatoma rubrovaria* Blanchard, 1843, exhibit well-known 4 chromatic morphotypes [23]. Other studies report the occurrence of natural homoploid hybrids between *T. infestans* and *Triatoma platensis* Neiva, 1913*, T. infestans* and *Triatoma rubrovaria*, and sympatric species of Phyllosoma complex and species of the *T. brasiliensis* complex. In addition, several hybrid species have been obtained experimentally [24]. This interspecific crossing can be decisive in originating and diversifying wild species, resulting in important epidemiological consequences due to differential competence and the capacity of hybrid vectors [3, 10, 11]. Therefore, the characterization (or identification) of Triatominae specimens based only on morphological and chromatic patterns, the most common identification method, is more complex than previously believed.

Studies on immature stages are crucial for group systematics. However, literature reports on immature forms of certain groups are scarce, difficult to use, or nonexistent. Many species undergo changes in color, structure, and morphology during their development, hindering their identification [25, 26]. Triatominae nymphs at this development stage are difficult to identify. To that end, molecular analyses are successfully used to characterize morphotypes of species complexes such as *T. brasiliensis *sp., which exhibits wide chromatic and morphological variation [7, 8].

Marked differences in color pattern and ecological features among species from the *Triatoma brasiliensis *complex were detected by microsatellites, mitochondrial *12S,* and *cytochrome b *genes, reinforcing species diagnosis [7, 16]. However, individuals from subspecies *T. b. brasiliensis* and *T. b. macromelasoma* might be clustered within the same *CO1* clade, since earlier studies have shown that some *cytochrome b* haplotypes of *T. b. macromelasoma* are similar to those of *T. b. brasiliensis* [16]. Moreover, these subspecies produce fertile hybrids when crossed in laboratory [3].

The basal clade of the Maximum Likelihood tree (Figure 1) was identified as subspecies *T. b. macromelasoma* because it is highly divergent (6%, as shown in the Nucleotide Distance Matrix, Table 2) from the other populations and was collected in its type locality [16]. On the other hand, all populations in the second clade were identified as *T. b. brasiliensis*. Their genetic similarity may be related to geographic proximity and similar habitat conditions. However, interpopulation divergence values (<1%) suggest that *T. b. brasiliensis *is still diversifying and/or exhibiting ongoing gene flow, probably due to human-assisted dispersal. Based on wing morphometry, Costa et al. [11] recently formulated a hypothesis that *T. b. macromelasoma* is the result of homoploidal hybridization between *T. b. brasiliensis* and *T. juazeirensis* in the state of Pernambuco, and that this is a form of speciation in sympatric populations.

In northeastern Brazil, the epidemiological importance of Triatominae bugs is mainly defined by their high rate of natural *T. cruzi* infection and ability to adapt to multiple ecotopes. Control measures therefore require a precise identification of which species of the *T. brasiliensis* complex is being targeted. Moreover, it is important to understand the ecoepidemiology of Triatominae since these vectors are found in large numbers in their natural habitat [27]. In this respect, the PCR-RFLP protocol described here is suggested as rapid, relatively simple, and economical assay to distinguish *Triatoma b. macromelasoma* from *Triatoma b. brasiliensis* subspecies. Even at small geographic scales, domestic populations are genetically structured by ecological parameters, thereby exhibiting small differences from the wild counterparts from which they are derived [8]. The present study highlights the effectiveness of the *CO1* gene in identifying subspecies of the *T. brasiliensis* complex and its contribution to classic taxonomy.

## Figures and Tables

**Table 1:** *Triatoma  brasiliensis* collection sites, coordinates, labels of selected sequences, and GenBank access numbers.

Localities (state)	Coordinates	Label	GenBank
Salgueiro (PE)	08°04′21.60′′S 39°07′57.39′′W	15 A/B	JQ088297/JQ088298
Monteiro (PB)	07°53′29.40′′S 37°07′00.79′′W	30 A/B	JQ088299/JQ088300
Mãe d'água (PB)	07°15′10.03′′S 37°25′58.25′′W	35 A/B	JQ088301/JQ088302
Lagoa Grande (PE)	08°59′07.10′′S 40°18′20.46′′W	41 A/B	JQ088303/JQ088304
Caicó (RN)	06°27′30.69′′S 37°06′09.71′′W	42 A/B	JQ088305/JQ088306
Serra Talhada (PE)	07°59′09.46′′S 38°17′37.70′′W	43 A/B	JQ088307/JQ088308
São José (PB)	06°52′00.00′′S 38°38′00.00′′W	53 A/B	JQ088309/JQ088310
Santa Cruz (PB)	06°31′33.48′′S 38°03′23.52′′W	54 A/B	JQ088311/JQ088312
São Francisco (PB)	06°36′53.43′′S 38°05′21.89′′W	55 A/B	JQ088313/JQ088314

**Table 2:** Matrix of divergence of *CO1* gene fragment of *T. brasiliensis* specimens using K2P model.

	1	2	3	4	5	6	7	8	9	10	11	12	13	14	15	16	17	18	19
(1) 15A Salgueiro PE																			
(2) 15B Salgueiro PE	0.000																		
(3) 30A Monteiro PB	0.039	0.039																	
(4) 30B Monteiro PB	0.039	0.039	0.000																
(5) 35A M$\tilde{\text{a}}$e d'$\overset{´}{\text{a}}$gua PB	0.037	0.037	0.001	0.001															
(6) 35B M$\tilde{\text{a}}$a d'$\overset{´}{\text{a}}$gua PB	0.037	0.037	0.001	0.001	0.000														
(7) 41A Lagoa Grande PE	0.031	0.031	0.013	0.013	0.011	0.011													
(8) 41B Lagoa Grande PE	0.031	0.031	0.013	0.013	0.011	0.011	0.000												
(9) 42A Caic$\overset{´}{\text{o}}$ RN	0.037	0.037	0.001	0.001	0.000	0.000	0.011	0.011											
(10) 42B Caic$\overset{´}{\text{o}}$ RN	0.037	0.037	0.001	0.001	0.000	0.000	0.011	0.011	0.000										
(11) 43A Serra Talhada PE	0.039	0.039	0.003	0.003	0.001	0.001	0.013	0.013	0.001	0.001									
(12) 43B Serra Talhada PE	0.039	0.039	0.003	0.003	0.001	0.001	0.013	0.013	0.001	0.001	0.000								
(13) 53A São Jos$\overset{´}{\text{e}}$ PB	0.037	0.037	0.001	0.001	0.000	0.000	0.011	0.011	0.000	0.000	0.001	0.001							
(14) 53B São Jos$\overset{´}{\text{e}}$ PB	0.037	0.037	0.001	0.001	0.000	0.000	0.011	0.011	0.000	0.000	0.001	0.001	0.000						
(15) 54A Santa Cruz PB	0.037	0.037	0.001	0.001	0.000	0.000	0.011	0.011	0.000	0.000	0.001	0.001	0.000	0.000					
(16) 54B Santa Cruz PB	0.037	0.037	0.001	0.001	0.000	0.000	0.011	0.011	0.000	0.000	0.001	0.001	0.000	0.000	0.000				
(17) 55A São Francisco PB	0.037	0.037	0.001	0.001	0.000	0.000	0.011	0.011	0.000	0.000	0.001	0.001	0.000	0.000	0.000	0.000			
(18) 55B São Francisco PB	0.039	0.039	0.003	0.003	0.001	0.001	0.013	0.013	0.001	0.001	0.003	0.003	0.001	0.001	0.001	0.001	0.001		
(19) AF021213.1 *Triatoma_sordida *	0.094	0.094	0.099	0.099	0.097	0.097	0.096	0.096	0.097	0.097	0.099	0.099	0.097	0.097	0.097	0.097	0.097	0.095	
(20) AF021186.1 *Triatoma_brasiliensis *	0.026	0.026	0.014	0.014	0.013	0.013	0.007	0.007	0.013	0.013	0.014	0.014	0.013	0.013	0.013	0.013	0.013	0.014	0.105
